# Glutamic acid decarboxylase 67 expression by a distinct population of mouse vestibular supporting cells

**DOI:** 10.3389/fncel.2014.00428

**Published:** 2014-12-17

**Authors:** Elisa Tavazzani, Simona Tritto, Paolo Spaiardi, Laura Botta, Marco Manca, Ivo Prigioni, Sergio Masetto, Giancarlo Russo

**Affiliations:** ^1^Department of Brain and Behavioral Sciences, University of PaviaPavia, Italy; ^2^Laboratory of Neurophysiology, Brain Connectivity Center, C. Mondino National Neurological InstitutePavia, Italy; ^3^Department of Biology and Biotechnology “L. Spallanzani”, University of PaviaPavia, Italy

**Keywords:** GAD67-GFP, hair cells, supporting cells, crista ampullaris, vestibular

## Abstract

The function of the enzyme glutamate decarboxylase (GAD) is to convert glutamate in γ-aminobutyric acid (GABA). Glutamate decarboxylase exists as two major isoforms, termed GAD65 and GAD67, that are usually expressed in GABA-containing neurons in the central nervous system. GAD65 has been proposed to be associated with GABA exocytosis whereas GAD67 with GABA metabolism. In the present immunofluorescence study, we have investigated the presence of the two GAD isoforms in the semicircular canal *cristae* of wild type and GAD67-GFP knock-in mice. While no evidence for GAD65 expression was found, GAD67 was detected in a distinct population of peripherally-located supporting cells, but not in hair cells or in centrally-located supporting cells. GABA, on the other hand, was found in all supporting cells. The present result indicate that only a discrete population of supporting cells use GAD67 to synthesize GABA. This is the first report of a marker that allows to distinguish two populations of supporting cells in the vestibular epithelium. On the other hand, the lack of GABA and GAD enzymes in hair cells excludes its involvement in afferent transmission.

## Introduction

Glutamic acid decarboxylase (GAD) catalyzes the conversion of L-glutamic acid to γ-aminobutyric acid (GABA) that in the central nervous system plays a critically important role in the inhibitory neurotransmission. Several studies indicate that in the adult brain, GAD exists as two isoforms, GAD65 and GAD67, where 65 and 67 refer to their respective molecular weights in kDa, which are encoded by two different independently regulated genes (Erlander et al., 1991; Bu et al., 1992). GAD65 is an amphiphilic, membrane anchored protein of 585 amino acid residues, while GAD67 is a cytoplasmic protein of 594 amino acid residues. There is 64% amino acid identity between the two isoforms, with the highest diversity located at the N terminus, which in GAD65 is required for targeting the enzyme to GABA-containing secretory vesicles. In neurons, GAD67 is spread evenly throughout the neuronal cytoplasm and it is believed that the GABA generated by GAD67 is utilized for purposes other than neurotransmission such as functioning as a trophic factor for synaptogenesis during early development, protection after neuronal injury, source of energy via the GABA shunt and regulator of redox potential during oxidative stress (Pinal and Tobin, 1998; Waagepetersen et al., 1999; Lamigeon et al., 2001). GAD65, on the other hand, is concentrated in the nerve terminals where it synthesizes GABA for neurotransmission purposes (Martin and Rimvall, 1993).

It is generally accepted that glutamate is the afferent transmitter at vestibular hair cell synapses (Ottersen et al., 1998; Bonsacquet et al., 2006). In contrast, the role of GABA in the mammalian vestibular system is controversial (for reviews see Guth et al., 1998; Soto et al., 2013).

Given the exclusive role of GAD65 and GAD67 in GABA synthesis, we have investigated the presence of these two enzymes in the semicircular canal *cristae* of wild type and GAD67-GFP knock-in mice, and their possible co-localization with GABA. While we found no expression of GAD65 in either supporting or sensory cells, GAD67 was found to be distinctively expressed by supporting cells of the peripheral, but not the central zone of the *crista*. On the other hand, GABA immunofluorescence was found in all supporting cells but not in hair cells. The functional implications of these results are discussed.

## Materials and methods

Experiments were performed on transgenic C57BL/6 GAD67-GFP knock-in heterozygous mice which were generated by Tamamaki and Yanagawa (Tamamaki et al., 2003) and were generously supplied by Prof. G. Biella (Pavia). Mice were sacrificed from postnatal day (P) 10 to P26. No differences in the GAD67 expression were seen among these ages, and data were therefore pooled. All experimental procedures involving animals were approved by the Ministero Italiano della Salute, and comply with the European international laws on animal research. Prior to any surgery, deep anesthesia was obtained by means of halothane (2-bromo-2-chloro-1,1,1-trifluoroethane).

**Figure 1:**
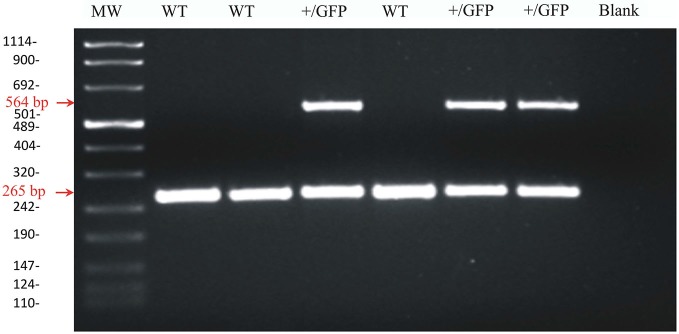
**Agarose gel image of PCR products for GAD67 and GAD67-GFP from wild-type and heterozygous GAD67-GFP knock-in mice**. Representative PCR products for GAD67 (265 pb) and GAD67-GFP (564 pb) were observed in +/GFP lanes (*n* = 3) while only the PCR product for GAD67 was present in the wild type lanes (WT, *n* = 3). The control lane (blank) was negative as expected. The left lane (MW) shows DNA molecular weight markers.

Transgenic mice were obtained by crossing female wild-type C57BL/6 mice with male heterozygous GAD67-GFP mice. Transgenic mice were sorted by two ways: (1) by examining the heads of P1-2 mice under a fluorescence lamp. GAD67-GFP knock-in mice exhibit a striking green fluorescence in the brain that can be visualized through the skull at this age; (2) by extracting DNA from mouse tails and carrying out the PCR. In the latter case, genomic DNA was extracted from mouse tail biopsies with the PureLink® Genomic DNA Midi Kit (Invitrogen, Italy). The extracted DNA was kept frozen at −80°C until use. PCR was performed on 2 μg DNA with the GoTaq® Flexi DNA Polymerase (Promega, Italy) and with specific primers for GAD67-GFP tagged mice (TR-1b 5′-GGCACAGCTCTCCCTTCTGTTTGC-3′; TR-3 5′-GCTCTCCTTTCGCGTTCCGACAG-3′; TRGFP-8 5′-CTGCTTGTCGGCCATGATATAGACG-3′). An initial denaturation at 94°C for 3 min was followed by 20 s at 96°C, 30 s at 68°C and 30 s at 72°C for 30 cycles. A final extension at 72°C for 10 min was performed. The molecular weight of the PCR products was compared to the DNA molecular weight marker VIII (Roche Molecular Biochemicals, Italy). The bands acquired with the Image Master VDS (Amersham Bioscience Europe, Germany) were at the expected size of 265 bp for GAD67 in wild type mice and of 265 bp and 564 bp for heterozygous GAD67-GFP mice (Figure 1).

### Semicircular canal crista preparation

Following deep anesthesia, mice were sacrificed by decapitation and the *ampullae* of the three semicircular canals (anterior, lateral and posterior) containing the *cristae* were rapidly dissected in an extracellular solution of the following composition (in mM): NaCl 135, CaCl_2_ 1.3, KCl 5.8, MgCl_2_ 0.9, HEPES 10, glucose 5.6, NaH_2_PO_4_ 0.7, Na-pyruvate 2, plus vitamins (10 ml/l MEM Vitamins Solutions 100x Gibco-Life Technologies, Italy) and aminoacids (20 ml/l MEM Amino Acids 50x Gibco-Life Technologies, Italy); pH 7.4 with NaOH.

The *ampullae* were then post-fixed in 4% paraformaldehyde solution (Sigma Aldrich, Italy) except for the experiments performed to detect GABA presence in which *ampullae* were post-fixed in 4% paraformaldehyde and 0.25% glutaraldehyde solution (Sigma Aldrich, Italy).

### Slice preparation for confocal microscopy

After 48 h, the fixed *ampullae* were embedded in 4% agar (Sigma-Aldrich, Italy) in extracellular solution, the agar blocks containing the *ampullae* were glued to the bottom of the Teflon plate of the vibroslicer chamber (Campden-Instrument, UK) filled with extracellular solution, and slices of the sensory epithelium of ~90 μm thickness were obtained.

Specimens were then mounted on slides for the confocal imaging. For the immunolabelling the specimens were washed with a 25% sucrose phosphate buffer solution (PBS), blocked for 60 min with 3% bovine serum albumin (Sigma Aldrich, Italy) in PBS and rinsed three times (5 min each) with PBS. Afterwards, the slides with the specimens were incubated overnight at 4°C with primary antibodies directed to calbindin (Calbindin D28K sc 7691 goat anti mouse, Santa Cruz Biotechnology, Italy) or to GAD65 (goat anti mouse 6113, Abcam, UK) or GABA antibody (guinea pig anti mouse ab17413 Abcam, UK), all diluted 1:100 in PBS. After three rinses in PBS (5 min each), the specimens treated with the primary antibodies were incubated (60 min at room temperature) with Alexa-fluor 633-secondary antibody conjugated (Life Technologies, Italy) or with Alexa fluor 594 secondary antibody (Life Technologies, Italy) at a dilution of 1:1000. The slides were then washed in PBS and mounted with Prolong Gold antifade reagent with DAPI (Invitrogen, Italy). Control experiments were performed simultaneously by omitting the primary antibody.

Fluorescence imaging was performed by a TCS SP5 II LEICA confocal microscopy system (Leica Microsystems, Italy) equipped with a LEICA DM IRBE inverted microscope. Images were acquired with 40X or 63X objectives and visualized by LAS AF Lite software (Leica Application Suite Advanced Fluorescence Lite version 2.6.0).

Since the vertical (anterior and posterior) canal *cristae* gave similar result, no differential analysis was performed.

## Results

**Figure 2:**
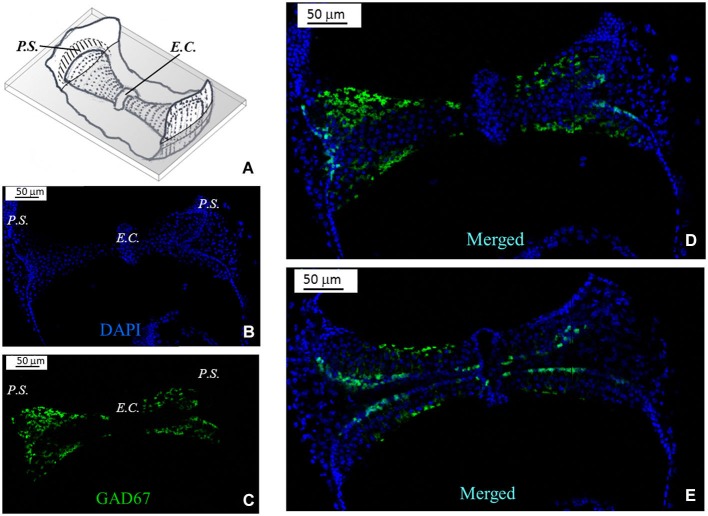
**GAD67 expression in the mouse vertical *crista*. (A)** Schematic representation showing a representative slice (gray) of the *crista ampullaris*. Dots represent hair bundles protruding from the *crista* surface. *E.C.*: *eminentia cruciata*; *P.S.*: *planum semilunatum*. **(B,C,D)** Photomicrographs of a superficial confocal section of the slice schematized in **(A)** showing the cell nuclei (blue; DAPI), the cellular expression of GAD67 (green; GFP), and the merged image, respectively. Light blue indicates co-localization of nuclei and GFP, which seems poor because GAD67 is expressed in the cytoplasm. Most cell bodies at this confocal level appear sectioned transversally.** (E)** Photomicrograph of a lower confocal section of the same specimen (merged image). Most cell bodies appear now sectioned longitudinally, as inferred from their elongated shape.

Because of the complex morphology of the semicircular canal *cristae*, we created schematic representations to illustrate the plane of the slices. A representative image is shown in Figure 2A for a slice obtained by cutting the vertical *ampulla* parallel to the surface of the *crista*. The most central region consists of the *eminentia cruciata* (*E.C.*), which in the mouse is about 50 μm long (*crista* longitudinal axis) and 100 μm large (*crista* transverse axis), contains one or two layers of non-sensory cells and is devoid of hair cells and afferent and efferent innervation (Purcell and Perachio, 1997; Desai et al., 2005). The lateral areas of the *crista* are bounded by the *planum semilunatum* (*P.S.*), a non-sensory epithelium of semilunar shape with a high cellular density. Since the *crista* curves along the walls of the *ampulla*, confocal scanning from top to bottom will at first show the most lateral regions only, and then the rest of the *crista*. Moreover, since sensory and supporting cells in the *crista* are fan-like arranged, depending on the level of the confocal scanning they will appear sectioned mostly transversally (upper sections) or longitudinally (lower sections).

**Figure 3:**
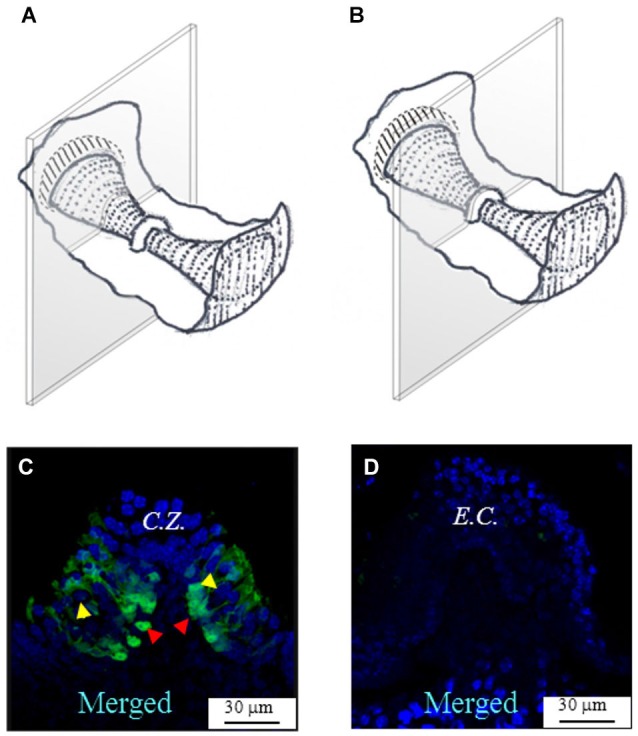
**GAD67 expression in transverse slices of the mouse vertical *crista*. (A,B)** Schematic representations showing the plane of the transverse sections. **(C,D)** Photomicrographs of the confocal sections showing the expression of GAD67. *C.Z.*: central zone. Note the absence of GAD67 at the most central zone in **(C)**, and the complete absence of GAD67 in the *E.C.* in **(D)**. The red arrowheads indicate the position of supporting cell nuclei, while the yellow arrowheads the position of hair cell nuclei.

**Figure 4:**
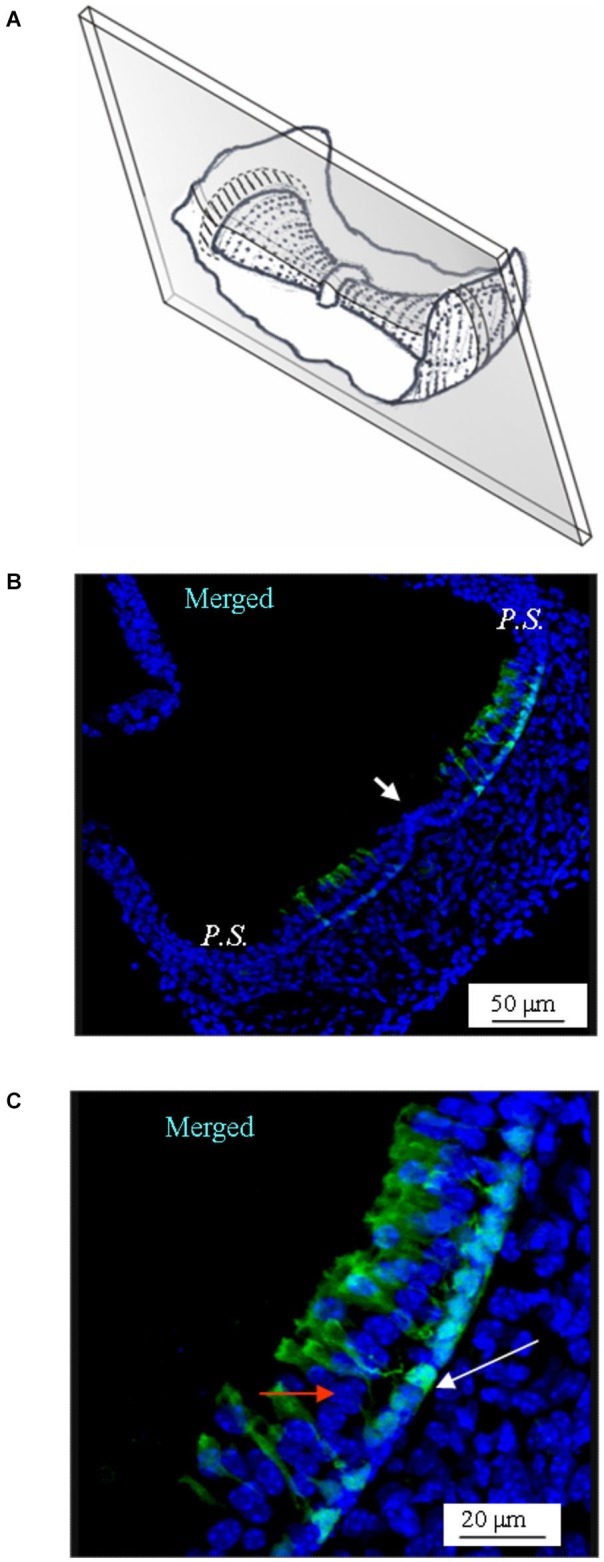
**GAD67 expression in longitudinal slices of the mouse vertical *crista*. (A)** Schematic representation showing the plane of the slice. Note that the section is not through the center of the *crista* (*i.e.,* it is not a medial section). **(B)** Merged photomicrograph showing the expression of GAD67. Note the absence of GAD67 expression in the *E.C.* (white arrow). **(C)** Enlargement of a portion of the same image as in **(B)**, showing in better detail the shape and position of the cells expressing GAD67. The white arrow points at the nuclear region of a supporting cell. The red arrow points at a nucleus of a hair cell.

Figure 2B shows a representative photomicrograph of an upper section of the slice cut as shown in Figure 2A. Here and in the next images cell nuclei are labeled with DAPI (blue). Note that GAD67 (Figure 2C, green) is expressed in the peripheral zone (*P.Z.*) of the *crista*, whereas no staining is present in the central zone (*C.Z.*) nor in the *E.C*. Merge of Figures 2B,C is shown in Figure 2D. Most cells at this section level are cut transversally. Figure 2E shows a photomicrograph of a lower section of the same specimen. Note that, despite the impression that stained cells are now dispersed throughout the *crista*, because of the complex *crista* morphology as discussed above, they are actually located in the *P.Z.* only. To confirm this, we also performed transverse and longitudinal slices. Figure 3 shows two representative transverse sections from a same vertical *crista*. Consistent with the previous images, GAD67 expression was not detectable in the *C.Z.* (Figure 3C) and in the *E.C.* (Figure 3D). At this magnification the shape and location of the GAD67-positive cells is also clear and typical of supporting cells—see also Figure 4C below. Note in fact their small nuclear regions (red arrowheads), which are aligned and in contact with the basement membrane, and their thin, thread-like bodies running in-between the sensory cells prior to enlarge at the apical (luminal) surface. In contrast, the nuclei of sensory hair cells (yellow arrowheads) occupy the upper layer and are slightly staggered to form a pseudostratified epithelium.

Similar results were obtained by sectioning the vertical *cristae* longitudinally (Figure 4). Note that since the slice was through the slope of the *crista* (i.e., not medial), the supporting cells located at both sides of the *E.C.* (arrowhead in Figure 4B) were GAD67-positive, which is consistent with Figures 2, 3. A magnification of the same image is shown in Figure 4C.

**Figure 5:**
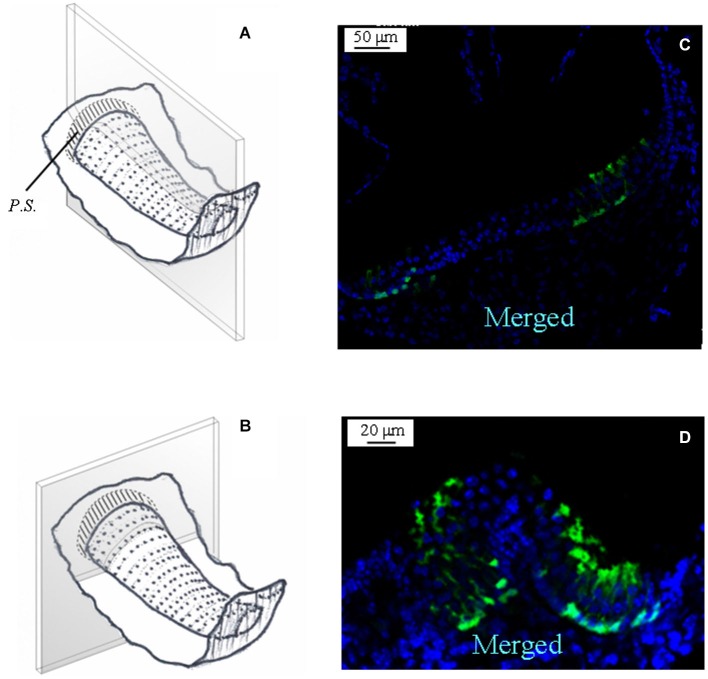
**GAD67 expression in the mouse horizontal *crista*. (A,B)** Schematic representations showing the plane of the longitudinal and transverse sections shown in **(C)** and **(D)**. Note the absence, also in the horizontal crista, of GAD67 expression in the central zone of the sensory epithelium.

Figure 5 shows the expression of GAD67 in a representative horizontal *crista*. Note that the morphology of the horizontal *crista* differs in not having the *E.C.* Like for the vertical *cristae*, however, GAD67 was found to be expressed by peripheral supporting cells only.

**Figure 6:**
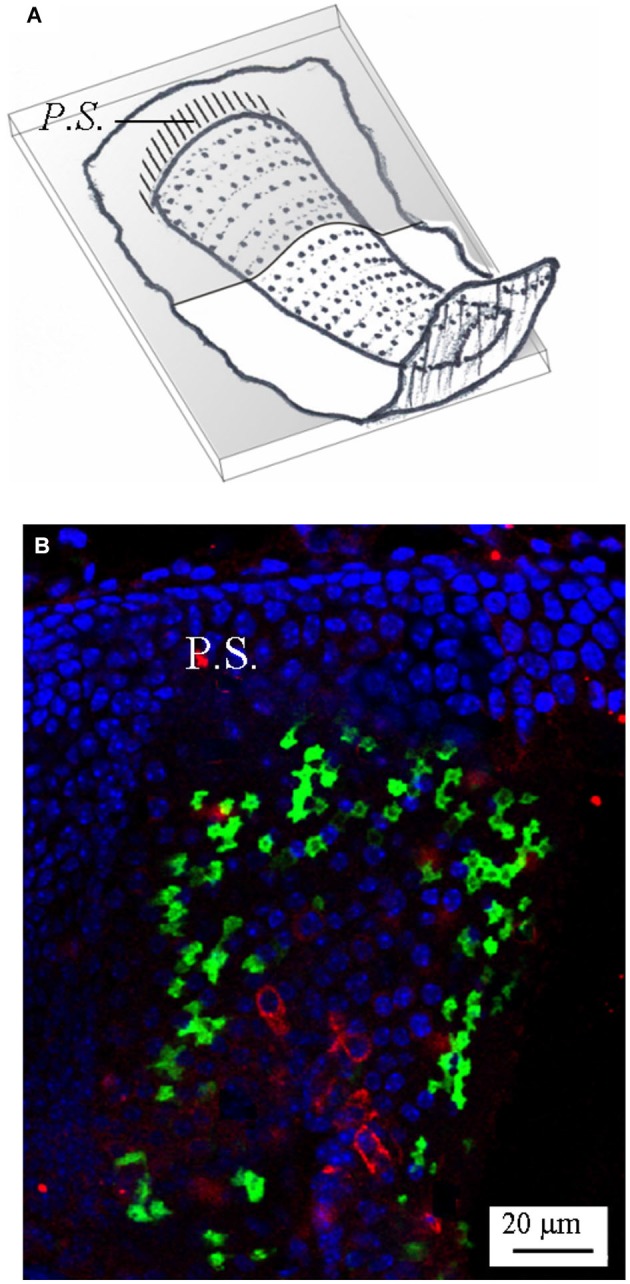
**Calbindin expression in the mouse horizontal *crista*. (A)** Schematic representation showing the plane of the section. **(B)** Photomicrograph of a longitudinal confocal section showing the cell nuclei (blue; DAPI), GAD67 (green; GFP), and calbindin (red; antibody) expression—merged image. Note that most calbindin immunolabeling is concentrated in the central zone, where it marks several calyx endings.

In some experiments, an antibody for calbindin was also used to show the calyx nerve terminals (Lysakowski et al., 2011). Figure 6 shows such an example in a horizontal *crista*; note that calbindin antibody (red) clearly stained several afferent calyces, mostly located in the central zone, where GAD67 was conversely absent.

**Figure 7:**
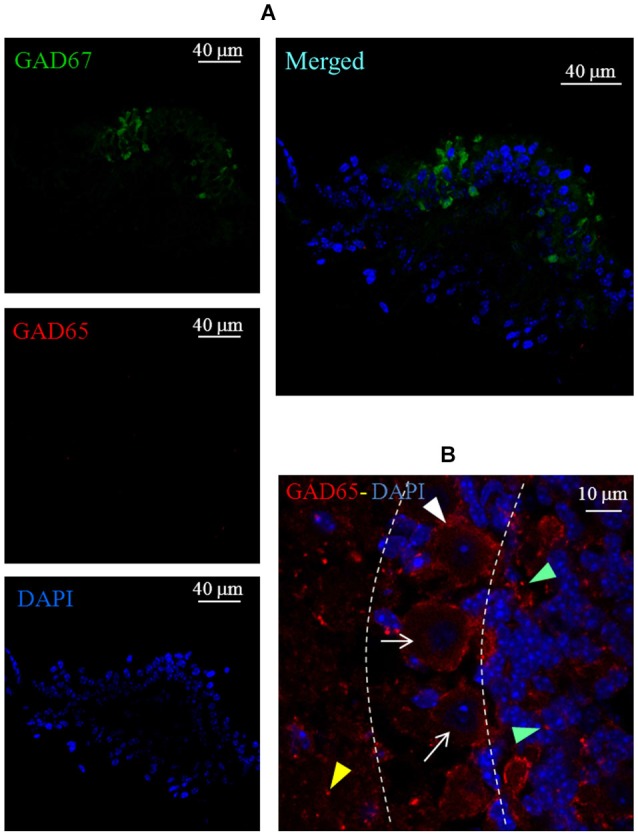
**Lack of GAD65 expression in the mouse *crista*. (A)** Photomicrographs of a *crista* transverse confocal section showing the expression of GAD67 (green), GAD65 (red), cell nuclei (blue; DAPI), and the merged image. Note the absence of GAD65 immunofluorescence. **(B)** Photomicrograph of a confocal section of a mouse cerebellum slice taken as positive control for GAD65 antibody labeling (red). Note the weak and diffuse immunofluorescence for GAD65 inside Purkinje cells (arrows) and immunofluorescent spots labeling around Purkinje cells (white arrowhead), in the molecular layer (yellow arrowhead) and in the granular layer (green arrowheads). Dashed lines indicate the bounders among the three layers.

A set of immunofluorescence labeling experiments was also performed to assess the expression of GAD65 in the *crista* sensory epithelium. However, we found no evidence for this isoform expression in either wild type (data not shown) or GAD67-GFP knock-in mice (Figure 7A). The functionality of the GAD65 antibody was previously assessed in the mouse cerebellum (Figure 7B).

**Figure 8:**
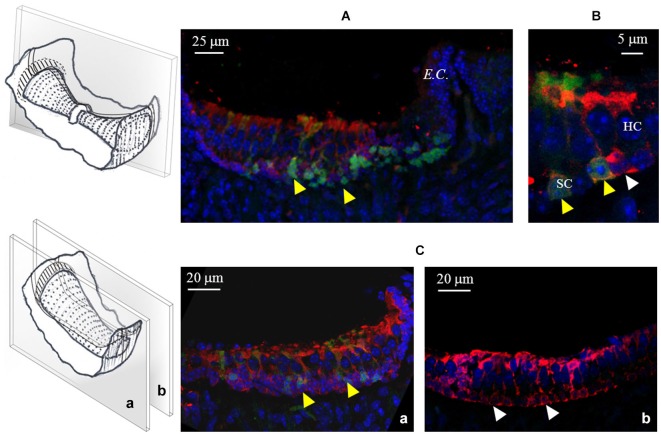
**GABA expression in the mouse *cristae*. (A)** Photomicrograph of a confocal section from a longitudinal slice of a vertical *crista*. As shown in the schematic representation on the left, the picture refers to the peripheral zone. Note the co-localization (yellow arrowheads) of GABA (red) and GAD67 (green) in many supporting cells. **(B)** Higher magnification from a different vertical *crista* section showing GABA and GAD67 co-localization (yellow arrowheads) or GABA-only expression (white arrowhead) in three different supporting cells. **(C)** Confocal images from two longitudinal slices of a horizontal *crista* depicting the peripheral **(a)** and the central **(b)** zone. Most peripheral supporting cells co-express GABA and GAD67, while central supporting cells only express GABA. No GABA expression was ever found in hair cells.

Finally, in order to test for the possible co-localization of GAD67 and GABA in the sensory *crista* epithelium, we performed a set of experiments in which GABA immunofluorescence was also tested in transgenic GAD67-GFP mice. As shown in Figure 8, GABA positivity was detected in all supporting cells, either expressing or not GAD67. No GABA expression was found in any hair cell.

**Figure 9:**
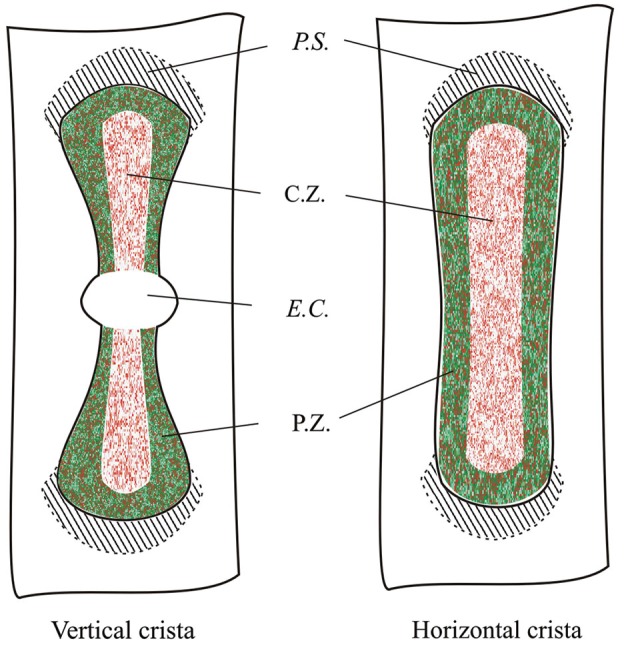
**Topographical distribution of GAD67 and GABA in the mouse vertical and horizontal *cristae***. Schematic representation showing the expression of GAD67 (green) by peripheral supporting cells as inferred by the confocal experiments. Red dots indicate supporting cell expressing GABA. C.Z.: central zone; P.Z.: peripheral zone; *P.S.*: *planum semilunatum*; *E.C.*: *eminentia cruciata*.

By analyzing several confocal images of vertical and horizontal canal *cristae*, we were able to reconstruct the topographical distribution of GAD67- and GABA-expressing supporting cells, which is shown in Figure 9.

## Discussion

Adult animals express two isoforms of GAD, GAD67 and GAD65. Both GAD isoforms can synthesize GABA, but GAD67 might preferentially synthesize cytoplasmic GABA for metabolic purposes while GAD65 for vesicular release (Soghomonian and Martin, 1998). The present study provides the first evidence for the expression in mouse vestibular *crista* of GAD67, whereas GAD65 was not found. GAD67 expression was confined to peripherally-located supporting cells, which also expressed GABA. The co-expression of GABA and GAD67 suggests a role for this enzyme in converting glutamate released from hair cells into GABA for its metabolic oxidation.

### GAD and GABA expression in crista sensory epithelium

In the mouse, there are about 800 Type I hair cells, 700 Type II hair cells and 1900 supporting cells *per crista* (Desai et al., 2005). Supporting cells play a major role in controlling glutamate concentration in the interstitial space following its release from sensory hair cells and its diffusion out of the synaptic cleft. Efficient clearance of glutamate is required to keep sensitivity of the system thus ensuring high-fidelity information transfer. This is particularly important in the vestibular sensory system because there is a continually high rate of neurotransmitter release, which is positively or negatively regulated depending on the direction of stereocilia deflection (head rotation) (Goldberg and Fernández, 1971). Consistent with their hypothesized role, supporting cells express the excitatory aminoacid transporter (EAAT) 1, also called GLAST (Ottersen et al., 1998). By showing that supporting cells express GAD67, the present study suggests that at least part of glutamate, once carried inside the supporting cell, is converted to GABA. GABA was in fact found to be present in supporting cells. However, since only peripheral supporting cells were found to express GAD67, and no expression was found for GAD65, the question arises of the presence of GABA in central supporting cells. One possibility could be that additional forms of GAD exist in the *crista*. Recently, it has been reported that the same mRNA coding for GAD67 can generate 10 splicing isoforms, one of which produces an enzymatically active 44 kDa peptide (GAD44) (Trifonov et al., 2014). Also, excess GABA synthetized by peripheral supporting cells might be transferred to central supporting cells by plasma membrane GABA transporters (Ito et al., 2007; Roth and Draguhn, 2012) expressed by all supporting cells.

### Functional meaning of GAD67 and GABA expression

The role of GABA in the vestibular epithelium has been matter of debate (Guth et al., 1998; Meza, 1998), and its possible involvement in vestibular signaling modulation remains to be ascertained. Our results indicate that neither GABA nor GAD are expressed by vestibular hair cells and thus it is possible to exclude a GABA involvement in afferent transmission. Here we found that all supporting cells, but not sensory cells, contain GABA. Moreover, GAD67, but not GAD65, was expressed in some supporting cells. In GABAergic neurons, GAD67 is found throughout the cell, whereas GAD65 preferentially localizes to synaptic terminals (Esclapez et al., 1994). Consistent with their subcellular localization, GAD67 provides both the cytoplasmic pool of GABA, which enters the tricarboxylic acid cycle to produce energy, and the vesicular pool of GABA (Tian et al., 1999; Lau and Murthy, 2012), while GAD65 primarily regulates the vesicular pool (Kaufman et al., 1991; Soghomonian and Martin, 1998), especially under conditions of sustained synaptic activity (Tian et al., 1999). Since vestibular supporting cells do not show a presynaptic machinery or vesicles, it seems reasonable to suggest that glutamate, once picked up by supporting cells, is converted into GABA for metabolic purposes. On the other hand, the possibility that GAD67 is also involved in some form of non-vesicular modulation (Soghomonian and Martin, 1998; Ito et al., 2007) of vestibular signaling can not be excluded.

### Why do only peripheral supporting cells express GAD67?

A major difference between the *P.Z.* and the *C.Z.* is the afferent innervation, as most complex calyces and calyx-only afferents are found in the *C.Z.* (Wersall, 1956; Lysakowski and Goldberg, 1997; Desai et al., 2005; see Figure 6 here), while bouton-only afferents are exclusively present in the *P.Z.* (Leonard and Kevetter, 2002). Bouton endings contact Type II hair cells, whereas calyx endings surround almost the entire basolateral surface of Type I hair cells. While in the case of Type II hair cells, clearance of glutamate involves its diffusion to supporting cells, in the case of Type I hair cells glutamate might be cleared from the calyx synaptic cleft by EAAT4 and EAAT5, two glutamate transporters recently found in Type I hair cells *and* calyx endings (Dalet et al., 2012). If glutamate released by Type I hair cells is cleared by EAAT4 and EAAT5, and glutamate released by Type II hair cells is cleared by GLAST expressed by the supporting cells, then why do only supporting cells located in the *P.Z.* express GAD67 given that Type II hair cells are also present in the *C.Z.*? One possible reason for the peripheral-only expression of GAD67 is that afferent nerve fibers innervating the *P.Z.* are characterized by a tonic discharge of action potentials, implying a continuous release of neurotransmitter, compared to the irregular firing of afferents from the *C.Z.* (Eatock and Songer, 2011). Glutamate, once picked up by supporting cells, can be converted into glutamine by glutamine-synthetase (Takumi et al., 1997). GAD67 could represent an additional mechanism for a more efficient metabolism of glutamate where its exocytosis is most abundant. It is important to consider that the rapid clearance of glutamate is required not only to restore the sensitivity of the system, but also to avoid glutamate cytotoxicity.

In conclusion, this is the first report of a marker that allows to distinguish peripheral supporting cells from central supporting cells in mammalian vestibular epithelia. This finding might be important for studies aimed at tracing cells lineage during vestibular epithelia regeneration, which is thought to occur by supporting cell transdifferentiation and/or mitosis (Rubel et al., 2013).

## Conflict of interest statement

The authors declare that the research was conducted in the absence of any commercial or financial relationships that could be construed as a potential conflict of interest.
