# Corrigendum: Glutamic acid decarboxylase 67 expression by a distinct population of mouse vestibular supporting cells

**DOI:** 10.3389/fncel.2015.00101

**Published:** 2015-04-01

**Authors:** Elisa Tavazzani, Simona Tritto, Paolo Spaiardi, Laura Botta, Marco Manca, Ivo Prigioni, Sergio Masetto, Giancarlo Russo

**Affiliations:** ^1^Department of Brain and Behavioral Sciences, University of PaviaPavia, Italy; ^2^Laboratory of Neurophysiology, Brain Connectivity Center, C. Mondino National Neurological InstitutePavia, Italy; ^3^Department of Biology and Biotechnology “L. Spallanzani”, University of PaviaPavia, Italy

**Keywords:** GAD67-GFP, crista ampullaris, hair cells, supporting cells, vestibular

In the below paragraph we refer to Lysakowski et al. (2011) for the use of calbindin, while the right reference has to be Leonard and Kevetter (2002).

In some experiments, an antibody for calbindin was also used to show the calyx nerve terminals (Leonard and Kevetter, 2002 instead of Lysakowski et al., 2011). Figure 6 shows such an example in a horizontal crista; note that calbindin antibody (red) clearly stained several afferent calyces, mostly located in the central zone, where GAD67 was conversely absent.

## Conflict of interest statement

The authors declare that the research was conducted in the absence of any commercial or financial relationships that could be construed as a potential conflict of interest.

